# Supplementary material for: Post-earthquake dizziness syndrome following the 2016 Kumamoto earthquakes, Japan
Source: PLoS One. 2021 Aug 5;16(8):e0255816. doi: 10.1371/journal.pone.0255816 (PMC8341659; doi:10.1371/journal.pone.0255816)
Supplement: S1 Table — (DOCX) [file pone.0255816.s001.docx]

**S1 Table. Patients’ demographic information**

| **Age category (years)** | **Males (n, %)** | **Females (n, %)** |
| --- | --- | --- |
| 10–12 | 205, 46.8 | 233, 53.2 |
| 13–15 | 642, 53.9 | 547, 46.1 |
| 16–18 | 324, 31.5 | 704, 68.5 |
| 19–29 | 27, 27.0 | 73, 73.0 |
| 30–39 | 75, 32.8 | 153, 67.2 |
| 40–49 | 98, 44.3 | 123, 55.7 |
| 50–59 | 89, 49.4 | 91, 50.6 |
| 60–69 | 9, 16.6 | 45, 83.4 |
| 70–79 | 18, 13.7 | 113, 86.3 |
| 80–89 | 10, 7.6 | 66, 92.4 |
| 90–100 | 1, 9.0 | 10, 91.0 |
