# Supplementary material for: Post-earthquake dizziness syndrome following the 2016 Kumamoto earthquakes, Japan
Source: PLoS One. 2021 Aug 5;16(8):e0255816. doi: 10.1371/journal.pone.0255816 (PMC8341659; doi:10.1371/journal.pone.0255816)
Supplement: S2 Table — (DOCX) [file pone.0255816.s002.docx]

**S2 Table**. **PEDS questionnaire**

| 1 | After the earthquake on Apr 14 and 16, have you felt dizziness as if you were swaying, despite there being no earthquake (PEDS) at that time? |
| --- | --- |
| 2 | Were you previously prone to experiencing motion sickness or seasickness? |
| 3 | Have you previously experienced vertigo/dizziness? |
| 4 | After the earthquake on Apr 14 and 16, have you experienced tinnitus and/or ear fullness? |
| 5 | After the earthquake on Apr 14 and 16, have you experienced anxiety? |
| 6 | After the earthquake on Apr 14 and 16, have you experienced autonomic symptoms (i.e., sweating abnormalities, digestive difficulties, urinary problems, and vision problems)? |
| 7 | What kind of dizziness did you experience: vertigo, the feeling of vibrating laterally, the feeling of the ground shaking, light-headedness, or motion sickness? |
| 8 | What was the date when you first experienced dizziness? When did that feeling stop? Immediately, a few hours, days, weeks, or months after the earthquake? |
| 9 | Did you feel dizzy indoors or outdoors? |
| 10 | What type of building were you in: iron reinforced building or wooden building? Was the building resistant to earthquakes or did it have base isolation? What floor were you on? |
