# Supplementary material for: Post-earthquake dizziness syndrome following the 2016 Kumamoto earthquakes, Japan
Source: PLoS One. 2021 Aug 5;16(8):e0255816. doi: 10.1371/journal.pone.0255816 (PMC8341659; doi:10.1371/journal.pone.0255816)
Supplement: Attachment — Submitted filename: renamed_57833.docx [file pone.0255816.s003.docx]

Reviewers' comments:

Reviewer's Responses to Questions

**Comments to the Author**
Reviewer #1: This paper outlines a cross-sectional analysis of post-earthquake dizziness syndrome (PEDS) following the 2016 series of tremors in Kumamoto, Japan. Results showed that more than one third of participants reported post-earthquake dizziness. Several factors were identified as related to PEDS, including female sex, ear fullness, an age of 21 or more, and anxiety. The authors conclude that earthquakes affect inner-ear function, autonomic symptoms, and psychological factors, both directly and indirectly via changes in environmental factors.

The paper offers a useful improvement to our knowledge about post-earthquake symptoms. The principal concern is that I would like to see a greater explanation of the mechanistic link between tremors and the outcomes measured here. A possible error in reporting statistics emerged (this is potentially a minor issue). Several other improvements could be made, as described below.

The Discussion is brief and leaves the reader with some lingering questions. As in the Introduction, the hypothesis linking earthquakes with lingering dizziness surely needs to be discussed at greater length. The statement by the authors “we examined sensory conflict theory and motion sickness” is true only to a very limited extent, this could be significantly expanded. I advise the authors to consider articles on postural stability and motion sickness (e.g. Stoffregen et al. 2013; Walton et al., 2011) as there seems to be a natural link between this research and the current study.
Response: I appreciate your valuable comments. I have added the description of motion sickness by postural stability theory in the Discussion.

Could the authors please check that they are correctly stating Pearson r values for their correlations? A negative correlation should be associated with a negative r value, but L137 states a positive value (note, degrees of freedom should also be added).

Response: Thank you for your valuable comment. I have corrected it and added degrees of freedom.

The authors asked participants to report “Autonomic symptoms” in their questionnaire. It must be clarified in the text what was specifically meant by “autonomic symptoms” and how they were described to participants.
Response: Thank you for your kind advice. I have added specific examples in the main text and Table S2.

It seems appropriate to reference the findings of a recent overview paper with clinical patients conducted by the main author (Miwa, 2021).
Response: Thank you for the suggestion. I have added the information accordingly.

L23 delete “the”
Response: Thank you for the suggestion. I have deleted it accordingly.

L34 I would press the authors to say more about their hypothesis. They state a multisensory mismatch produces PEDS, but this is descriptive and not explanatory. How could a single-shot mismatch result in PEDS, or are aftershocks likely to be a necessary factor? Are there existing models that support this hypothesis?
Response: I appreciate your comment. I have added the required information to the Introduction.

L50 It would be valuable to state that these participants reported no head trauma brought about by the earthquake. I am assuming that those data were obtained. If they were not collected, this could be added as a limitation.
Response: Thank you. I have added the description regarding this in the Materials and Methods section.

L69 “changes in the proportion of patients with PEDS … were compared by hypothesis testing” This does not specify the type of test used (e.g. t-tests), which should be included here.
Response: Thank you for your comment. I have corrected it accordingly.

L74 Could the authors specify what proportion of the responses were imputed?
Response: Yes. The values of building type were imputed because some building occupants did not know to live in ironed-type buildings or wooden-type buildings.

L87 The number of participants in this study should be stated in the ‘Participants’ section.
Response: Thank you for your comment. I have stated the number of participants in the ‘Participants’ section.

L122 The authors could briefly state here what the non-significant factors were (e.g. “Building type and regional location had no significant association with PEDS”).
Response: Thank you for your comment. I have added the required information to the revised manuscript.

L133 A caveat here is that the correlation contains very few datapoints, and this should be stated by the authors in text, not a figure caption (e.g. “While the correlation analysis contained only 5 datapoints and is thus limited in statistical power, visual inspection supports the idea that no relationship exists between aftershocks and PEDS”).
In addition, Table 3 references “earthquake sickness ratio”, I assume this should be corrected to PEDS. Column 1 in Table 3 can also be removed, with ‘Kumamoto Region’ being the heading of column 2.
Response: Thank you for your valuable comments. I have added the suggested description in the main text. In addition, I have corrected “earthquake sickness ratio” to “PEDS”, following your suggestion. In Table 3, column 1 was removed, and I added ‘Kumamoto region’ in the heading of column 2.

L135 “Despite the negative correlation between geological conditions/number of aftershocks and prevalence of PEDS” I would call this a ‘null’ correlation, not a negative one.
Response: Thank you. I have corrected accordingly.

I advise adding significance asterisks in (e.g.) Fig 2b.
Response: Thank you for the valuable advice. According to your suggestion, I have added significance asterisks in Fig 2b.

References
Miwa, T. (2020). Vestibular function after the 2016 Kumamoto earthquakes: a retrospective chart review. Frontiers in Neurology, 11.
Stoffregen, T. A., Chen, F. C., Varlet, M., Alcantara, C., & Bardy, B. G. (2013). Getting your sea legs. PLoS One, 8(6), e66949.
Walton, D., Lamb, S., & Kwok, K. C. (2011). A review of two theories of motion sickness and their implications for tall building motion sway. Wind and Structures, 14(6), 499.

Munafo, J., Wade, M. G., Stergiou, N., & Stoffregen, T. A. (2015). Subjective reports and postural performance among older adult passengers on a sea voyage. Ecological Psychology, 27, 127-143.

Reviewer #2: The examination of post-earthquake effects on perception of balance and movement is an interesting phenomenon that the authors were able to take advantage of after the Kumamoto earthquake. The data collection and analysis were well done. However, it is not clear why the authors are couching this a sensory mismatch issue. That is why is this the theoretical mechanism that is thought to account to the phenomenological data they collected. There needs to be a clear justification of why mismatch perspectives are the right model to use. Without that connection there isn't a strong justification for the research questions. Are there other alternatives that could also account for your findings?

Response: Thank you for your valuable comments. I have expanded the Discussion section and reconsidered adding the theory on motion sickness (i.e. postural instability theory and sensory conflict theory cited from Stoffregen et al. 2013; Walton et al., 2011). In this study, I speculated that both theories were associated with PEDS. I have added them in the Discussion section. (Lines 181-191)
