# Supplementary material for: Post-earthquake dizziness syndrome following the 2016 Kumamoto earthquakes, Japan
Source: PLoS One. 2021 Aug 5;16(8):e0255816. doi: 10.1371/journal.pone.0255816 (PMC8341659; doi:10.1371/journal.pone.0255816)
Supplement: Attachment — Submitted filename: renamed_faf31.docx [file pone.0255816.s004.docx]

Reviewer #1: I am confused as to why the authors include only one to two sentences on the mechanism linking earthquake with sickness (i.e. L120-126).

Please be aware, many readers will not be familiar with the idea that sensory conflict or postural instability are thought to cause motion sickness. As a result, it is worthwhile to take the time and explain those ideas in some more detail here. Not too much--this is clearly not the focus of the paper--but I would expect more than the manuscript currently contains.

Response: Thank you for your valuable comments. I have made sure that in the revision, these concepts are explained with enough detail that readers will be sufficiently informed.

Indeed, the Discussion contains some of this relevant information that belongs in the Introduction. L273-297 describes previous literature and theory details that I would expect to read before the hypothesis is stated. Please consider re-phrasing this information and transposing it to the Introduction. It can then be referred to in the Discussion: How do your results fit with this literature?

Response: We appreciate your suggestion and I have rephrased the literature information and transposed it into the Introduction. In addition, I made sure to point them out and integrate them into our results in the discussion section. I have considered that stimulation via environment, such as an earthquake, a ship, a building facing wind, causes various degrees of motion sickness.

Related to this point, the authors' new inclusions regarding sensory conflict theory and motion sickness were not clear. For example, "In addition, numerous repetitive aftershocks caused low dosage motion sickness via the postural instability theory/ecological approach to perception and action." (L317). There is insufficient evidence for the authors to make this claim as it is written, without qualifiers ("One explanation of our results is that..."). It is also unclear what is the 'ecological approach to perception and action' to a non-expert reader, so it seems appropriate to delete those last 6 words.

Response: Thank you for your comments. I agree with the assessment made by the reviewer and have deleted the 'ecological approach to perception and action'. Further, I have added the qualifier "One explanation of our results is that..." in this sentence. My intention was to get across that the mechanisms of motion sickness were different whether it was instigated by the major earthquake or the repetitive aftershocks.

It is ultimately true that the authors cannot make any strong statements about what was the mechanistic cause for their results (sensory conflict/postural stability), as their study was not designed to test any one theory. So, they should make this point clear in their limitations. Please note, it is not a strong limitation: This is a very interesting paper, even if it cannot speak to the mechanisms at play in motion sickness.

Response: I greatly appreciate your kind advice. I have added this concern to the limitation section.

Remove 'this correlation was not significant' (L248).

Response: Thank you for the advice, I have removed this statement.

L300-301: "motion sickness":Perhaps this should this state "prior history of motion sickness"?

Response: Your suggested wording does better reflect our intended meaning. I have revised this statement throughout the manuscript.

The authors should review the the newly added material as there are several language mistakes.

Response: I apologize for this oversight. We have edited the manuscript to ensure it meets adequate English language standards.

Reviewer #2: You have thoughtfully addressed the initial issues raised by the reviewers - the intent of my particular comments was not to make you change your theory argument, just justify it. However, I think your revisions on this issue are reasonable. My only suggestion is to make sure to be clear that postural/ecological perspectives on motion sickness do not support sensory conflict explanations. Phenomenologically, the participants experiences might suggest conflict(which is where I think you were going originally) - but as you noted, definitive claims cannot be made at this point.

Response: Thank you for valuable comments. After your suggestion, I have reconsidered the mechanisms underlying earthquake-related dizziness, and concluded that the major earthquake and the repetitive aftershocks caused motion sickness via different mechanisms. I have described this in the manuscript. However, as you noted, the stated mechanism is just speculation. As such, I have also mentioned this in the limitation section.
